# Gastric bypass surgery in morbid obesity: Influence on thyroid function tests and insulin resistance status

**DOI:** 10.22088/cjim.15.2.294

**Published:** 2024

**Authors:** Parvin Layegh, Razieh Ayati, Ali Jangjoo, Mohammad Ali Yaghoubi

**Affiliations:** 1 Metabolic Syndrome Research Center, Mashhad University of Medical Sciences, Mashhad, Iran; 2 Department of Internal Medicine, Faculty of Medicine, Birjand University of Medical Sciences and Health Services, Birjand, Iran; 3 Surgical Oncology Research Center, Mashhad University of Medical Sciences, Mashhad, Iran

**Keywords:** Gastric bypass surgery, Euthyroid, thyroid function tests, Insulin resistance.

## Abstract

**Background:**

There are inconsistent results about the effect of gastric bypass surgery on thyroid function tests in morbidly obese subjects. The aim of this study was to investigate the changes in thyroid function tests and insulin resistance status in euthyroid morbidly obese subjects before and three months after gastric bypass surgery (GBS).

**Methods:**

Twenty-nine subjects with morbid obesity (BMI≥40) were enrolled in this before-after study. Patients with known thyroid disorders or a history of thyroid ablative therapy, users of drugs that affect thyroid function, or fasting blood sugar and insulin were excluded. TSH, Free T4, total T3, fasting blood sugar and insulin level, and BMI were measured before and 3 months after GBS. Statistical analysis was performed with appropriate tests and p<0.05 was considered significant.

**Results:**

Body mass index (BMI), insulin sensitivity index (HOMA-IR), and total T3 significantly decreased after bypass surgery (all with p<0.001) but no significant changes were seen in TSH (P=0.203) and FreeT4 (P=0.33). There was a significant negative correlation between changes in HOMA-IR and changes in FreeT4 (P=0.038, r= -0.38). There was no statistically significant correlation between the percentage of excess BMI loss (%EBMIL) and changes in T3 (P=0.66), Free T4 (P=0.92), TSH (P= 0.27), and HOMA-IR (P=0.17).

**Conclusion:**

Although significant changes can occur in BMI, insulin sensitivity index, fasting blood sugar, and T3 in short-time follow-up after bariatric surgery, significant TSH and FreeT4 changes may need longer follow-ups.

Morbid obesity is an important health problem that affects the thyroid gland as a main organ that involves in the regulation of body energy balance. Although some believe that obesity is the result of long-term thyroid dysfunction (1, 2), others attribute thyroid hormonal changes to weight gain and obesity per se (3). Insulin resistance and hyperinsulinemia due to obesity lead to hyperleptinemia which in turn induces an elevation in serum TSH. Additionally, high inflammatory cytokines in obesity lead to a decrease in iodide absorption (4). 

There are controversies not only about the association between thyroid function tests (TFT) and BMI (Body Mass Index) but also about changes in thyroid hormonal profile after treatment of obesity with nutritional interventions or bariatric surgery (5-13). The current study was designed to compare thyroid hormonal profiles before and three months after bariatric surgery in a cohort of subjects with morbid obesity (BMI≥ 40). We also evaluated the association between changes in thyroid function tests and insulin resistance status in our participants. 

## Methods

In the present study, we evaluated 29 subjects with morbid obesity (BMI≥40) before and three months after gastric bypass surgery. Patients with known thyroid disorders or a history of thyroid ablation therapy, users of drugs that affect thyroid function or fasting blood sugar (FBS), and insulin levels were excluded. TSH, FreeT4, Total T3, FBS, and insulin were measured and HOMA-IR (Homeostatic Model Assessment for insulin resistance) and BMI were calculated before and three months after gastric bypass surgery (GBS). Glucose was measured by an enzymatic colorimetric (GOD-PAP) method using Biorexfars kits (Iran) with an intra-assay CV of 1.37% and inter-assay CV of 1.51%. The assay range of glucose in adults was 70-115 mg/dl. T3 was measured by ADVIA Centaur CP System T3 assay (Siemens) with an assay range of 0.1-8 ng/ml. FreeT4 was measured by ADVIA Centaur CP T4 assay (Siemens) with an assay range of 0.1-12.0 ng/dl and a normal range of 0.89-1.76 ng/dl. TSH was measured by ADVIA Centaur CP TSH assay (two-site sandwich immunoassay using chemiluminometric technology and with an assay range of 0.01-150 mU/L. Insulin was measured by a solid-phase enzyme-labeled chemiluminescent immunometric assay (IMMULITE 2000XPI) (Siemens) with an analytical sensitivity of 2 μu/ml. HOMA-IR was calculated by using this formula: HOMA-IR= ((glucose (mg/dl) × insulin (μU/ml))/405. Also, BMI and excess BMI loss (EBMIL) % were calculated using these formulas: 

BMI=weight (kg)/[height (m)]^2^

And 

EBMIL%: [(Preoperative BMI- current BMI)/ (preoperative BMI- 25)] ×100

A checklist was used for gathering the demographic data. A paired-t test and Wilcoxon sign-rank test were used to compare the preoperative and the three months postoperative values in our patients. The correlation between variables was assessed by Pearson’s or Spearman’s Rho tests. Written Informed consent was obtained from all participants. The study was approved by the Research Ethics Committee of Mashhad University of Medical Sciences (IR.MUMS.Medical.REC.1398.407). Statistical analyses were performed with SPSS Version 26.0 and p<0.05 was considered statistically significant.

## Results

A total of 29 patients were enrolled in this study; eleven of whom (37.9%) were males and 18 (62.1%) were females. The mean age of participants was 38.52±8.57. The variable values before and after surgery and the amount of their changes are summarized in table 1. Although the increase in Free T4 after surgery was not statistically significant, there was a significant negative correlation between changes in HOMA-IR and changes in Free T4 in this study (P=0.038, r=- 0.38). The mean±SD of EBMIL was 50.89±17.37% and its median (IQR) was 47.04% (37.47-64.07%). The minimum and maximum EBMIL were 24.83% and 86.41%, respectively. 44.82% of participants had EBMIL more than or equal to 50% and 55.18% had EBMIL of less than 50%. There was no statistically significant correlation between EBMIL and changes in T3 (P=0.66), Free T4 (P=0.92), TSH (P=0.27), and HOMA-IR (P=0.17). 

**Table 1:** Variable values before and three months after gastric bypass surgery

**P- value**	**Change**		**After surgery**		**Before surgery**		**Variables**
	**Median** **(IQR)**	**Mean±SD**	**Median** **(IQR)**	**Mean±SD**	**Median** **(IQR)**	**mean±SD**	
<0.001	8.84 (7.82-12.67)	10.84±5.29	34.90 (40.78-48.36)	35.28±5.20	43.90 (40.78-48.36)	45.93±6.26	**BMI (kg/m** ^2^ **)** *
<0.001	16.10 (6.95-21.85)	14.57±7.68	6.50 (4.15-10.60)	8.13±7.03	20.70 (16.05-28.90)	22.71±10.38	**Insulin(μu/ml)**
<0.001	10 (3.50-17)	9.48±9.41	88 (82.50-94)	89.06±9.84	97 (91.50-103)	98.55±9.99	**FBS(mg/dl)**
<0.001	3.60 (2.15-5.65)	3.70±1.91	1.30 (0.85-2.20)	1.85±1.78	4.80 (3.65-6.85)	5.55±2.64	**HOMA-IR**
0.203	0.30 (-0.45-1.10)	0.32±1.32	1.90 (1.30-2.65)	2.09±1.09	2.10 (1.50-3.15)	2.41±1.07	**TSH(μu/ml)**
0.332	0.0 (-0.17-0.09)	-0.03±0.18	1.30 (1.16-1.38)	1.26±0.14	1.20 (1.06-1.40)	1.23±0.19	**FreeT4(ng/dl)**
<0.001	14 (4.75-31.50)	16.46±21.62	109 ((97.50-123)	111.01±15.52	128 (105-146.50)	127.48±23.99	**Total T3(ng/dl)**

* by Wilcoxon test, others by paired t-test

Body mass index (BMI), fasting blood sugar (FBS), Homeostatic Model Assessment for Insulin Resistance-insulin resistance (HOMA-IR), and thyroid stimulation hormone (TSH)

## Discussion

In the present study, BMI, insulin, fasting blood sugar, HOMA-IR and total T3 decreased significantly three months after surgery but, no statistically significant change was seen in TSH level. Also, we found a non-significant increase in FreeT4 level. Although a significant negative correlation was seen between changes in HOMA-IR and changes in FreeT4, there was no correlation between changes in EBMIL and Free T4. 

Twelve months after bypass surgery, Neves et al. (9) found a significant decrease in TSH in euthyroid patients with morbid obesity when basal TSH was high-normal (TSH≥2.5 mu/l), but this finding was not seen in patients with basal TSH less than 2.5 mU/L. In contrast to our results, Paula Juiz-Valiña (10) found a significant decrease in TSH and FreeT4 levels in obese patients, 12 months after gastric bypass surgery. The decrement in TSH was progressive over time after surgery and was significantly associated with EBMIL. A significant decrease in TSH level that was parallel with weight loss and a decrease in BMI was reported in Gokosmanoglu et al. investigation (14). Similar results were also found in the study of Fangyuan (15), six months after R&Y gastric bypass in euthyroid patients with obesity and type 2 diabetes. 

In a meta-analysis by Guan et al (16), changes in T3 and Free T4 were similar to our study but inconsistently they found a significant decrease in TSH levels after surgery. In line with the current study, Dall'Asta et al. (17) showed a significant decrease in T3 and no change in TSH level after bypass surgery. In Zendel's (18) investigation, a decrease in TSH and no change in T4 was seen after bypass surgery. Zhang (19) followed-up with patients up to 36 months after surgery and showed a significant decrease in FT3, and FT4 and no change in TSH. Similar to our study, Maccuish (20) showed no change in TSH but a significant increase in FreeT4 in subjects with morbid obesity.

It seems that a decrease in deiodinase D1 and D2 results in an increase in FT4 and a decrease in FreeT3 (21, 22) and changes in TFT are due to adaptation with body weight changes (16). In our study decrease in insulin and HOMA-IR was seen as similar to other investigations (23, 24, and 25). Changes in BMI give rise to an increase in glucagon-like peptide 1 (GLP-1) that may influence insulin physiology (26, 27). Also, a decrease in insulin may influence weight loss (28). In our study, there was a significant negative correlation between FreeT4 changes and changes in insulin resistant index that is similar to Farasat al. (29). Different duration of follow-up between the present study and the studies that were mentioned above may somewhat explain the different results in levels of TFT. Also, progressive weight loss over time and its effect of it may somewhat explain the absence of significant change in TSH level in our short-time study.

Similar to the TSH subgroup with TSH less than 2.5 in Neves^,^ study (9), in the present study the mean TSH before surgery was less than 2.5 mU/L, and no significant decrease in TSH level was seen in three months after surgery. 

Paula Juiz-Valiña et al. (10) showed that 12 months after bariatric surgery the EBMIL was 72.7±2.1% and a decrement in TSH level over time was associated with the EBMIL. Again, short-time follow-up in our study may influence not only the changes in TSH and freeT4 levels but also the correlation between changes in these parameters and EBMIL after bariatric surgery. 

Also, the use of iodine/selenium supplements, patients^,^ iodine and selenium status before bypass surgery, and their adherence to use these compounds after surgery may affect thyroid function tests. This may partially explain the different results of various studies that addressed this issue. Finally, the small sample size in our study limited the performance of the subgroup analysis. Also, no normalization of TSH values for seasonal variations was done. It is obvious that the increase in sample size and longer duration of follow-up can influence the results of similar investigations that may be done in the future. Although in short-time follow-up after bariatric surgery, significant changes can occur in BMI, insulin sensitivity index, fasting blood sugar and T3 level, the occurrence of significant changes in TSH and FreeT4 may need longer follow-ups. 
